# Variable selection in multivariate multiple regression

**DOI:** 10.1371/journal.pone.0236067

**Published:** 2020-07-17

**Authors:** Asokan Mulayath Variyath, Anita Brobbey

**Affiliations:** Department of Mathematics and Statistics, Memorial University of Newfoundland, St. John’s, NL, Canada; Tongii University, CHINA

## Abstract

**Introduction:**

In many practical situations, we are interested in the effect of covariates on correlated multiple responses. In this paper, we focus on estimation and variable selection in multi-response multiple regression models. Correlation among the response variables must be modeled for valid inference.

**Method:**

We used an extension of the generalized estimating equation (GEE) methodology to simultaneously analyze binary, count, and continuous outcomes with nonlinear functions. Variable selection plays an important role in modeling correlated responses because of the large number of model parameters that must be estimated. We propose a penalized-likelihood approach based on the extended GEEs for simultaneous parameter estimation and variable selection.

**Results and conclusions:**

We conducted a series of Monte Carlo simulations to investigate the performance of our method, considering different sample sizes and numbers of response variables. The results showed that our method works well compared to treating the responses as uncorrelated. We recommend using an unstructured correlation model with the Bayesian information criterion (BIC) to select the tuning parameters. We demonstrated our method using data from a concrete slump test.

## Introduction

Multivariate multiple regression analysis is often used to assess covariate effects when one or multiple response variables are collected in observational or experimental studies. Many multivariate regression techniques are designed for univariate responses. A common way to deal with multiple response variables is to apply the univariate technique separately to each variable, ignoring the joint correlation among the responses.

**Fig 1:**
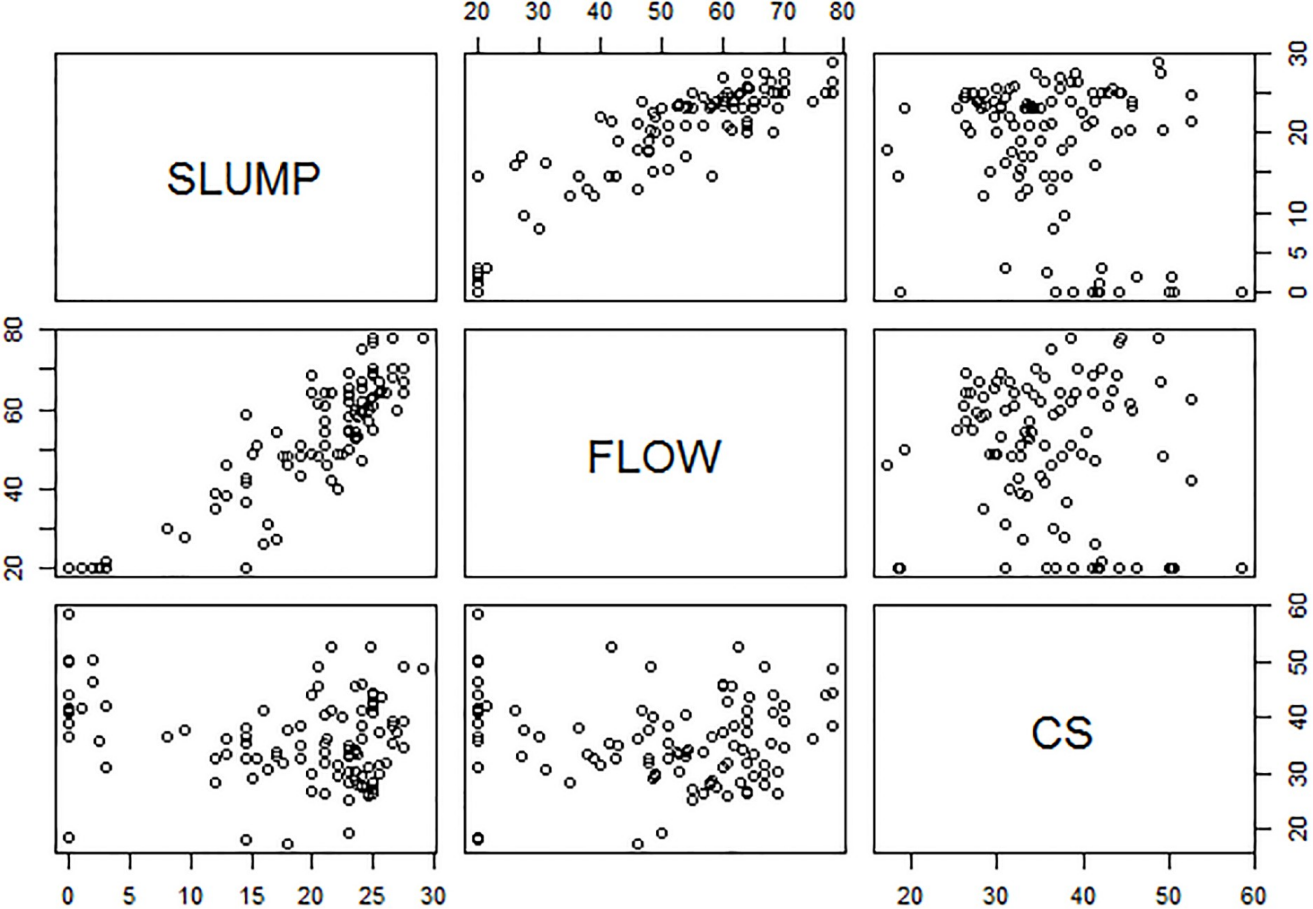
Scatter plot indicating the relationship between slump (*Y*_1_), flow (*Y*_2_), and compressive strength (*Y*_3_).

Consider the concrete slump test study reported in [1], [2], and [3]. The data set consists of three continuous output variables (slump, flow, and 28-day compressive strength (CS)). We wish to model these responses as a function of seven concrete ingredients (covariates): cement (*X*_1_), fly ash (*X*_2_), blast furnace slag (*X*_3_), water (*X*_4_), super plasticizer (*X*_5_), coarse aggregate (*X*_6_), and fine aggregate (*X*_7_). The responses are correlated, and separate regression analysis will not take into account the importance of covariance on the response variables. Fig 1 shows the correlation among the output variables; in particular, slump and flow are highly correlated.

The joint model for all responses results in 27 parameters that must be estimated. Some of the covariates have no influence on the response variable(s), and excluding them results in a simpler model with better interpretive and predictive value.

The multi-response regression problem has been studied by various researchers in the generalized linear model (GLM) framework. For instance, the curd and whey method [4] uses the correlation among the response variables to improve the predictive accuracy. Multivariate modeling methods have been extensively used in transportation and accident analysis, especially for binary outcomes [5–7]. Some researchers have explored multivariate modeling with consideration of correlation in a Bayesian framework. For example, see [8] for the modeling of multivariate spatio-temporal Tobit regression and [9] for an approach based on spatial analysis.

The analysis of multivariate outcomes is more difficult when there are multiple types of outcomes. These occur frequently in the investigation of, e.g., dose–response experiments in toxicology [10, 11], birth defects in teratology [12], and pain in public health research [12, 13]. The methodologies used for mixed outcomes include factorization-based approaches on extensions of the general location model [14, 15]. However, these approaches depend on parametric distributional assumptions. Approaches based on latent variables include [16] and [17]. Modified generalized estimating equations (GEEs) [18] have been used to model longitudinal data; these approaches are of great interest because of their simplicity.

The GEE approach of [18] provides flexible modeling of multivariate observations based on a quasi-likelihood (QL) approach. In QL modeling, one assumes the existence of the first two moments of the responses of interest. It extends the GEE methodology to simultaneously analyze binary, count, and continuous outcomes with nonlinear models that incorporate the intra-subject correlation. The method uses a working correlation matrix. The incorporation of the intra-subject correlation makes this approach attractive. However, when we apply a joint model for all responses, many regression parameters must be estimated, and some have little or no influence on the responses. Large models can be difficult to interpret, so variable selection for multi-response modeling is of great interest.

We first systematically study the GEE approach in a cross-sectional set-up with multiple responses [11, 19]. Simultaneous parameter estimation and variable selection [20] has been used in many areas, including longitudinal data analysis [21]. We have extended this method to multivariate multiple regression using a penalized GEE methodology. We use the Bayesian information criterion (BIC) and generalized cross validation (GCV) to find the tuning parameters. Our simulation studies show that our methodology performs well.

The remainder of the paper is organized as follows. In the next section, we review the GEE for multiple responses and introduce our penalized GEE and the computational procedures. We discuss the distributional properties of the estimates and presents the simulation studies, subsequently and provides concluding remarks in the last section.

## Materials and methods

### GEE for multiple outcomes

We now discuss the GEE model based on the marginal distributions of the response for the analysis of longitudinal data. In a cross-sectional study with multiple responses, [12] used the GEE approach to estimate the parameters. Let the observations $\left(y_{i}^{m},x_{i}^{m}\right)$ denote the response and covariate respectively for the *m*th response (*m* = 1, 2, …, *M*_*i*_) measured on subject *i* = 1, …, *n*. The QL approach requires us to specify the first two moments of the data ($y_{i}^{m}$). We define

$$\begin{array}{c} E\left(y_{i}^{m}\right)=\mu_{i}^{\left(m\right)}=f\left(x_{i}^{m},\beta^{\left(m\right)}\right) \end{array}$$

$$var\left(y_{i}^{\left(m\right)}\right)=s^{\left(m\right)}h^{\left(m\right)}\left(\mu_{i}^{\left(m\right)}\right)=\sigma_{i}^{2(m)}$$

where *h*^(*m*)^(⋅) is a known function, *s*^(*m*)^ is a scaling parameter, *f*^(*m*)^(⋅) is a nonlinear function of the coefficients, and ***β***^(*m*)^ is a *p*^(*m*)^ × 1 vector of model coefficients for the *m*th response variable. Let $y_{i}=\left(y_{i}^{\left(1\right)},\ldots ,y_{i}^{\left(M_{i}\right)}\right),\mu_{i}=\left(\mu_{i}^{\left(1\right)},\ldots ,\mu_{i}^{\left(M_{i}\right)}\right)$ and $\beta ={\left(\beta^{{\left(1\right)}^{T}},\beta^{{\left(2\right)}^{T}},\ldots ,\beta^{{\left(M\right)}^{T}}\right)}^{T}$ be the *p* × 1 vector of model parameters for all *M* outcomes, where *p* = (*p*^(1)^ + *p*^(2)^ + ⋯ + *p*^(*M*)^). In the QL framework with multiple outcomes, the regression coefficients ***β*** can be estimated by solving the GEEs

$$\begin{array}{c} S\left(\beta \right)=\sum_{i=1}^{n}D_{i}^{T}V_{i}^{-1}\varepsilon_{i}=0. \end{array}$$ (1)

For each subject *i*, let **D**_*i*_ be an *M*_*i*_ × *p* full-rank derivative diagonal block matrix such that ${\text{D}}_{i}=\text{diag}\left(\frac{\partial \mu_{i}^{\left(1\right)}}{\partial \beta^{{\left(1\right)}^{T}}},\frac{\partial \mu_{i}^{\left(2\right)}}{\partial \beta^{{\left(2\right)}^{T}}},\cdots ,\frac{\partial \mu_{i}^{\left(M_{i}\right)}}{\partial \beta^{{\left(M_{i}\right)}^{T}}}\right)$, ***ε***_***i***_ = (***y***_***i***_ − ***μ***_***i***_) be an *M*_*i*_ × 1 vector of residuals, and $V_{i}=A_{i}^{1/2}R_{i}\left(\alpha \right)A_{i}^{1/2}$ be the *M*_*i*_ × *M*_*i*_ working covariance matrix of ***y***_***i***_. Here, $A_{i}=\text{diag}\left(\sigma_{i}^{2(1)},\sigma_{i}^{2(2)},\ldots ,\sigma_{i}^{2(M_{i})}\right)$ is an *M*_*i*_ × *M*_*i*_ diagonal matrix of $\text{var}\left(y_{i}^{\left(m\right)}\right)$ and ***R***_***i***_(***α***) is an *M*_*i*_ × *M*_*i*_ working correlation matrix parameterized with the parameter vector ***α***. The GEE estimator $\hat{\beta }$ is asymptotically consistent as *n* goes to infinity.

### Penalized GEE

To perform parameter estimation and variable selection simultaneously in the presence of mixed discrete and continuous outcomes, we propose a penalized version of the extended GEEs [12, 19]. Penalized likelihood methods such as LASSO [22] and SCAD [20] have been successful both theoretically and in practice. All the variables are considered at the same time, which may lead to a better global submodel. The penalized GEE has the feature that the consistency of the model holds even if the working correlation is misspecified. However, to improve the statistical efficiency of the coefficient, we recommend a covariance matrix based on the estimate of the unstructured working correlation. The regression coefficients ***β*** can be estimated by solving the penalized GEEs defined by

$$\begin{array}{c} \sum_{i=1}^{n}D_{i}^{T}V_{i}^{-1}\varepsilon_{i}-nP_{\lambda }^{\prime }\left(\beta \right)sign\left(\beta \right)=0 \end{array}$$ (2)

where $P_{\lambda }^{\prime }\left(\beta \right)=\partial P_{\lambda }\left(\beta \right)/\partial$ ***β*** is the vector derivative of the penalty function *P*_λ_(*β*) with λ being the vector of tuning parameters.

Although different penalty functions can be adopted, we consider only LASSO and SCAD. The former has the sparsity property, and the latter simultaneously achieves the three desirable properties of an ideal penalty: sparsity, unbiasedness, and continuity [20]. The LASSO penalty defined as *P*_λ_(*β*) = ||*β*|| as per [22], where as per [20], the derivative of the SCAD penalty is defined as

$$\begin{array}{c} P_{\lambda ,a}^{\prime }\left(\beta \right)=\lambda \{I\left(\beta \leq \lambda \right)+\frac{{\left(a\lambda -\beta \right)}_{+}}{(a-1)\lambda }I\left(\beta >\lambda \right)\}\;for\;some\;a>2\;and\;\beta >0. \end{array}$$

where *a* and λ are tuning parameters.

#### Computational algorithm

To compute $\hat{\beta }$, we use the local quadratic approximation (LQA) algorithm [20]. With the aid of the LQA, the optimization of (2) can be carried out using a modified Newton–Raphson (MNR) algorithm. The estimate of $\hat{\beta }$ at the (*r* + 1)th iteration is

$$\begin{array}{c} {\hat{\beta }}_{r+1}={\hat{\beta }}_{r}-\{\frac{\partial S({\hat{\beta }}_{r})}{\partial \beta }-n\Sigma_{\lambda }\left({\hat{\beta }}_{r}\right){\}}^{-1}\{S\left({\hat{\beta }}_{r}\right)-nU_{\lambda }\left({\hat{\beta }}_{r}\right)\} \end{array}$$ (3)

where

$$\begin{array}{c} \Sigma_{\lambda }\left(\beta_{r}\right)=\text{diag}(P_{\lambda }^{\prime }\left(\right|\beta_{1r}\left|)/\right|\beta_{1r}|,\ldots ,P_{\lambda }^{\prime }\left(\right|\beta_{pr}\left|)/\right|\beta_{pr}\left|\right),\\ \frac{\partial S({\hat{\beta }}_{r})}{\partial \beta }=-\sum_{i=1}^{n}D_{i}^{T}V_{i}^{-1}D_{i};;\;\;\;U_{\lambda }\left(\beta_{r}\right)=\Sigma_{\lambda }\left(\beta_{r}\right)\beta_{r}. \end{array}$$

Given a tuning parameter λ, we repeat the above algorithm to update ${\hat{\beta }}_{r}$ until we achieve convergence.

#### Correlation structure

Many researchers (e.g., [23–25]) have shown that an incorrectly specified correlation structure reduces the estimation efficiency. Thus, we suggest using an unstructured correlation structure ***R***_***u***_(***α***) to estimate each variance and covariance uniquely. This structure can be estimated using a residual-based moment method. Let $\hat{V(\alpha )}={\hat{A}}^{1/2}diag\left({\hat{R}}_{u},\ldots ,{\hat{R}}_{u}\right){\hat{A}}^{1/2}$ be the unstructured covariance matrix estimate. We have

$$\begin{array}{c} {\hat{R}}_{u}=\frac{1}{n}\sum_{i=1}^{n}{\hat{A}}_{i}^{-1/2}\varepsilon_{i}\varepsilon_{i}^{T}{\hat{A}}_{i}^{-1/2}. \end{array}$$

### Tuning parameter selection

We set *a* = 3.7 for SCAD penalty as per [20]. Thus, we tune **λ** for both LASSO and SCAD. We define the GCV [26] criterion via

$$\begin{array}{c} GCV\left(\lambda \right)=\frac{1}{n}\frac{D}{{\left(1-n^{-1}df\left(\lambda \right)\right)}^{2}} \end{array}$$

and the BIC (see [27] and [6]) via

$$\begin{array}{c} BIC\left(\lambda \right)=log(\frac{D}{n})+(\frac{log(n)}{n})df\left(\lambda \right) \end{array}$$

where *D* is the deviance of the model and *df*(λ) = *tr*{*X*(*X*^*T*^ *X* + *n*Σ_λ_)^−1^ *X*^*T*^}. We choose the tuning parameter λ that minimizes GCV(λ) and BIC(λ).

### Properties of estimates

Let $\beta =(\beta_{A},\beta_{N})$ be the true vector of the regression coefficients. Under some necessary regularity conditions [28, 29] for sufficiently large *n*, the parameter estimates of the penalized GEE with the LASSO (λ = *O*_*p*_(*n*^−1/2^)) and SCAD (λ = *o*_*p*_(1)) penalties are consistent and asymptotically normal, i.e.,

$$\begin{array}{c} \sqrt{n}\left(\hat{\beta }-\beta \right)\overset{D}{\to }N\left(0,\Omega_{0}^{-1}\Omega_{1}\Omega_{0}^{-1}\right) \end{array}$$

where $\Omega_{0}^{-1}\Omega_{1}\Omega_{0}^{-1}$ is the sandwich variance estimator, with $\Omega_{0}=\sum_{i=1}^{n}D_{i}^{T}V_{i}^{-1}D_{i}-n\Sigma_{\lambda }\left(\hat{\beta }\right)$ and $\Omega_{1}=\sum_{i=1}^{n}D_{i}^{T}V_{i}^{-1}\text{cov}\left(y_{i}\right)V_{i}^{-1}D_{i}$.

The cov(y_i_) can be replaced by $\varepsilon_{i}\varepsilon_{i}^{T}$.

## Results

### Performance analysis

We conducted a series of simulation studies to investigate the performance of our variable selection approach on continuous, binary, and count response outcomes using the LASSO and SCAD penalty functions. The simulations were conducted using the R software. For faster optimization of the tuning parameter λ, we used the warm-starting principle, where the initial value of ***β*** is replaced by ${\hat{\beta }}_{\left(\lambda +\delta \lambda \right)}$ for the MNR algorithm. We select the model with minimum BIC(λ) or GCV(λ). We assess the model performance using the model error (ME) [20] as well as the standard error and the correct and incorrect deletions. The ME is due to the lack of fit of an underlying model and is denoted by $ME(\hat{\beta })$. Its size reflects how well the model fits the data:

$$\begin{array}{c} ME\left(\hat{\beta }\right)=E_{x}{\left\{\mu \left(X\beta \right)-\mu \left(X\hat{\beta }\right)\right\}}^{2} \end{array}$$

where *μ*(***X***|***β***) = *E*(***y***|**X**). The ME has been expressed as the median relative model error (MRME). The relative model error is defined via

$$\begin{array}{c} RME=\frac{ME}{ME_{full}}, \end{array}$$

where *ME*_*full*_ is the ME calculated by fitting the data with the full model. The correct deletions are the average number of true zero coefficients correctly estimated as zero, and the incorrect deletions are the average number of true nonzero coefficients erroneously set to zero. In the tables, the estimated values for correct and incorrect deletions are reported in the columns “Correct” and “Incorrect”. For comparison purposes, we estimated the covariance matrix of the response variables based on both the unstructured working correlation (UWC) and the independent working correlation (IWC). We simulated 1000 data sets consisting of *n* = 50 and *n* = 100 observations from the response model

$$\begin{array}{c} g\left(E\left(Y\right)\right)=X_{ij}^{T}\beta \end{array}$$

with *i* = 1, 2, … *n* subjects and *j* = 1, 2, …, *m* responses. For binary outcomes we use a logit link; for count outcomes we use a log link; and for continuous (normal) outcomes we use the identity link function. We generated the covariates *X*_*ij*_ from the multivariate normal distribution with marginal mean 0, marginal variance 1, and AR(1) correlation with *ρ*_*x*_ = 0.5. For the simulations, we considered the following three cases of continuous, binary, and count response outcomes with different ***β*** values and correlation *ρ*_*y*_ between the responses and with $\sigma_{y}^{2}=1$.

**Case 1: Three correlated cormal responses**. We consider correlated normal responses (*m* = 3) with AR(1) true correlation. We set *ρ*_*y*_ = 0.7 and consider two covariates (*k* = 2) with ***β*** = (***β***^(1)^, ***β***^(2)^, ***β***^(3)^) = ((3, 1.5), (0, 0), (2, 0)). The simulation results are summarized in Table 1 for IWC and Table 2 for UWC. The tables show that the nonzero estimates of both SCAD and LASSO are close to the true values, i.e., $\beta_{1}^{\left(1\right)}=3$, $\beta_{2}^{\left(1\right)}=1.5$, and $\beta_{1}^{\left(3\right)}=2$. However, the standard errors of the estimates in Table 2 are lower, which can be attributed to the correlation between the responses. For both *n* = 50 and *n* = 100, the mean ME and its standard error are smaller for SCAD than LASSO. The average number of zero coefficients increases as *n* increases in Table 2, especially for SCAD. This indicates that SCAD performs better than LASSO.

**Table 1:** Simulations results for correlated normal responses (Case 1) with IWC.

Selection	Penalty	MRME	Correct	Incorrect
*n* = 50				
${\hat{\lambda }}_{GCV}$	SCAD	0.064	1.297	0.000
	LASSO	0.092	0.982	0.001
${\hat{\lambda }}_{BIC}$	SCAD	0.053	1.532	0.000
	LASSO	0.113	1.180	0.002
*n* = 100				
${\hat{\lambda }}_{GCV}$	SCAD	0.030	1.298	0.000
	LASSO	0.038	0.871	0.001
${\hat{\lambda }}_{BIC}$	SCAD	0.025	1.538	0.000
	LASSO	0.043	1.066	0.000
Selection	Penalty	${\hat{\beta }}_{1}^{\left(1\right)}$	${\hat{\beta }}_{2}^{\left(1\right)}$	${\hat{\beta }}_{1}^{\left(3\right)}$
*n* = 50				
${\hat{\lambda }}_{GCV}$	SCAD	2.998(0.171)	1.496(0.168)	1.993(0.154)
	LASSO	2.898(0.203)	1.388(0.219)	1.831(0.229)
${\hat{\lambda }}_{BIC}$	SCAD	2.998(0.171)	1.496(0.168)	1.992(0.147)
	LASSO	2.866(0.236)	1.356(0.244)	1.789(0.266)
*n* = 100				
${\hat{\lambda }}_{GCV}$	SCAD	2.998(0.115)	1.506(0.116)	1.996(0.105)
	LASSO	2.931(0.170)	1.438(0.154)	1.891(0.152)
${\hat{\lambda }}_{BIC}$	SCAD	2.998(0.115)	1.506(0.115)	1.998(0.100)
	LASSO	2.898(0.216)	1.403(0.192)	1.857(0.190)

**Table 2:** Simulations results for correlated normal responses (Case 1) with UWC.

Selection	Penalty	MRME	Correct	Incorrect
*n* = 50				
${\hat{\lambda }}_{GCV}$	SCAD	0.045	1.457	0.000
	LASSO	0.079	1.214	0.001
${\hat{\lambda }}_{BIC}$	SCAD	0.035	1.661	0.000
	LASSO	0.079	1.261	0.011
*n* = 100				
${\hat{\lambda }}_{GCV}$	SCAD	0.022	1.513	0.000
	LASSO	0.040	1.265	0.000
${\hat{\lambda }}_{BIC}$	SCAD	0.017	1.696	0.000
	LASSO	0.040	1.318	0.000
Selection	Penalty	${\hat{\beta }}_{1}^{\left(1\right)}$	${\hat{\beta }}_{2}^{\left(1\right)}$	${\hat{\beta }}_{1}^{\left(3\right)}$
*n* = 50				
${\hat{\lambda }}_{GCV}$	SCAD	2.999(0.155)	1.496(0.145)	1.992(0.137)
	LASSO	2.884(0.200)	1.427(0.156)	1.842(0.185)
${\hat{\lambda }}_{BIC}$	SCAD	3.000(0.145)	1.496(0.131)	1.993(0.122)
	LASSO	2.861(0.212)	1.421(0.164)	1.823(0.236)
*n* = 100				
${\hat{\lambda }}_{GCV}$	SCAD	2.998(0.102)	1.505(0.098)	1.996(0.091)
	LASSO	2.921(0.122)	1.457(0.100)	1.892(0.125)
${\hat{\lambda }}_{BIC}$	SCAD	2.999(0.092)	1.504(0.090)	1.996(0.083)
	LASSO	2.917(0.122)	1.454(0.100)	1.887(0.124)

**Case 2: Two correlated normal responses and one independent binary response**. We consider three outcomes (*m* = 3): two continuous and one binary. The continuous outcomes were generated from a normal distribution and were correlated with AR(1) true correlation. We set *ρ*_*y*_ = 0.7 and consider the binary outcome from an independent binary observation and two covariates (*k* = 2) with ***β*** = (***β***^(1)^, ***β***^(2)^, ***β***^(3)^) = ((3, 1.5), (0, 0), (2, 0)). The simulation results are summarized in Tables 3 and 4. The tables show that the nonzero estimates for IWC are similar to those for UWC. However, because of the large correlation (0.7) between the continuous responses, the standard errors of $\beta_{1}^{\left(1\right)}=3$, $\beta_{2}^{\left(1\right)}=1.5$ are smaller for UWC. Again, the average number of zero coefficients is higher for UWC than for IWC. As the SCAD sample size increases, the mean ME and its standard error decrease for both GCV and BIC. The LASSO estimates for $\beta_{3}^{\left(1\right)}$ are not close to the true value, but the SCAD estimates of the nonzero coefficients are all close to the true values. Thus, SCAD performs better than LASSO.

**Table 3:** Simulations results for correlated normal and independent binary responses (Case 2) with IWC.

Selection	Penalty	MRME	Correct	Incorrect
*n* = 50				
${\hat{\lambda }}_{GCV}$	SCAD	0.059	1.755	0.007
	LASSO	0.129	1.663	0.024
${\hat{\lambda }}_{BIC}$	SCAD	0.054	2.143	0.030
	LASSO	0.154	1.787	0.051
*n* = 100				
${\hat{\lambda }}_{GCV}$	SCAD	0.027	1.816	0.001
	LASSO	0.072	1.799	0.023
${\hat{\lambda }}_{BIC}$	SCAD	0.023	2.122	0.003
	LASSO	0.095	2.002	0.043
Selection	Penalty	${\hat{\beta }}_{1}^{\left(1\right)}$	${\hat{\beta }}_{2}^{\left(1\right)}$	${\hat{\beta }}_{1}^{\left(3\right)}$
*n* = 50				
${\hat{\lambda }}_{GCV}$	SCAD	2.995(0.171)	1.494(0.165)	2.192(0.799)
	LASSO	2.888(0.188)	1.381(0.201)	0.772(0.423)
${\hat{\lambda }}_{BIC}$	SCAD	2.996(0.171)	1.494(0.165)	2.069(0.919)
	LASSO	2.864(0.204)	1.355(0.218)	0.687(0.419)
*n* = 100				
${\hat{\lambda }}_{GCV}$	SCAD	2.997(0.115)	1.506(1.113)	2.078(0.487)
	LASSO	2.906(0.145)	1.413(0.144)	0.903(0.435)
${\hat{\lambda }}_{BIC}$	SCAD	2.997(0.115)	1.506(0.113)	2.060(0.470)
	LASSO	2.876(0.159)	1.381(0.167)	0.731(0.383)

**Table 4:** Simulations results for correlated normal and independent binary responses (Case 2) with UWC.

Selection	Penalty	MRME	Correct	Incorrect
*n* = 50				
${\hat{\lambda }}_{GCV}$	SCAD	0.056	1.829	0.005
	LASSO	0.094	1.762	0.006
${\hat{\lambda }}_{BIC}$	SCAD	0.037	2.209	0.037
	LASSO	0.097	1.824	0.008
*n* = 100				
${\hat{\lambda }}_{GCV}$	SCAD	0.025	1.825	0.001
	LASSO	0.057	1.880	0.002
${\hat{\lambda }}_{BIC}$	SCAD	0.015	2.336	0.001
	LASSO	0.063	2.091	0.002
Selection	Penalty	${\hat{\beta }}_{1}^{\left(1\right)}$	${\hat{\beta }}_{2}^{\left(1\right)}$	${\hat{\beta }}_{1}^{\left(3\right)}$
*n* = 50				
${\hat{\lambda }}_{GCV}$	SCAD	2.995(0.156)	1.492(0.148)	2.192(0.815)
	LASSO	2.918(0.148)	1.429(0.141)	0.782(0.391)
${\hat{\lambda }}_{BIC}$	SCAD	2.998(0.142)	1.488(0.133)	2.076(0.936)
	LASSO	2.912(0.150)	1.424(0.140)	0.739(0.364)
*n* = 100				
${\hat{\lambda }}_{GCV}$	SCAD	2.999(0.108)	1.501(1.002)	2.079(0.480)
	LASSO	2.938(0.102)	1.453(0.094)	0.882(0.388)
${\hat{\lambda }}_{BIC}$	SCAD	3.002(0.096)	1.498(0.102)	2.066(0.469)
	LASSO	2.927(0.097)	1.445(0.091)	0.767(0.299)

**Case 3: Two correlated normal responses and one binary response**. We consider three outcomes (*m* = 3): two continuous and one binary. They are generated using an unstructured correlation structure with the parameters *ρ*_12_ = 0.3, *ρ*_13_ = 0.4, and *ρ*_23_ = 0.6, and we consider two covariates (*k* = 2) with ***β*** = (***β***^(1)^, ***β***^(2)^, ***β***^(3)^) = ((3, 1.5), (0, 0), (2/3, 0)). We set the ***β*** values for the binary outcome smaller than before to avoid numerical instability. The correlated normal and binary outcomes were generated in R using the **BinNor** package [30] for generating multiple binary and normal variables simultaneously given marginal characteristics and association structure; it is based on the methodology of [31]. The simulation results are summarized in Tables 5 and 6. The tables show that if the sample size is increased, the mean ME and its standard error are reduced. Again, the standard errors of the nonzero parameter estimates are lower for UWC than IWC. The average numbers of zero coefficients using SCAD with BIC for all sample sizes are close to the target value of three, and for SCAD with GCV the nonzero estimated coefficients are close to the true values for *n* = 50 and *n* = 100.

**Table 5:** Simulations results for correlated normal and binary responses (Case 3) with IWC.

Selection	Penalty	MRME	Correct	Incorrect
*n* = 50				
${\hat{\lambda }}_{GCV}$	SCAD	0.071	1.916	0.209
	LASSO	0.092	1.343	0.173
${\hat{\lambda }}_{BIC}$	SCAD	0.070	2.446	0.301
	LASSO	0.119	1.509	0.258
*n* = 100				
${\hat{\lambda }}_{GCV}$	SCAD	0.034	1.775	0.066
	LASSO	0.050	1.449	0.084
${\hat{\lambda }}_{BIC}$	SCAD	0.047	2.430	0.151
	LASSO	0.056	1.622	0.152
Selection	Penalty	${\hat{\beta }}_{1}^{\left(1\right)}$	${\hat{\beta }}_{2}^{\left(1\right)}$	${\hat{\beta }}_{1}^{\left(3\right)}$
*n* = 50				
${\hat{\lambda }}_{GCV}$	SCAD	2.997(0.167)	1.499(0.171)	0.543(0.520)
	LASSO	2.899(0.202)	1.395(0.214)	0.241(0.224)
${\hat{\lambda }}_{BIC}$	SCAD	2.997(0.167)	1.499(0.170)	0.246(0.461)
	LASSO	2.886(0.219)	1.361(0.238)	0.212(0.222)
*n* = 100				
${\hat{\lambda }}_{GCV}$	SCAD	2.998(0.114)	1.503(0.116)	0.633(0.201)
	LASSO	2.918(0.149)	1.421(0.157)	0.287(0.194)
${\hat{\lambda }}_{BIC}$	SCAD	2.998(0.113)	1.503(0.115)	0.309(0.432)
	LASSO	2.892(0.166)	1.393(0.188)	0.253(0.185)

**Table 6:** Simulations results for correlated normal and binary responses (Case 3) with UWC.

Selection	Penalty	MRME	Correct	Incorrect
*n* = 50				
${\hat{\lambda }}_{GCV}$	SCAD	0.065	1.975	0.167
	LASSO	0.098	1.538	0.117
${\hat{\lambda }}_{BIC}$	SCAD	0.059	2.493	0.242
	LASSO	0.106	1.601	0.241
*n* = 100				
${\hat{\lambda }}_{GCV}$	SCAD	0.031	1.980	0.041
	LASSO	0.059	1.578	0.057
${\hat{\lambda }}_{BIC}$	SCAD	0.037	2.537	0.094
	LASSO	0.063	1.700	0.079
Selection	Penalty	${\hat{\beta }}_{1}^{\left(1\right)}$	${\hat{\beta }}_{2}^{\left(1\right)}$	${\hat{\beta }}_{1}^{\left(3\right)}$
*n* = 50				
${\hat{\lambda }}_{GCV}$	SCAD	2.998(0.153)	1.496(0.153)	0.574(0.498)
	LASSO	2.883(0.178)	1.417(0.173)	0.209(0.237)
${\hat{\lambda }}_{BIC}$	SCAD	2.993(0.147)	1.495(0.145)	0.287(0.464)
	LASSO	2.872(0.180)	1.407(0.181)	0.190(0.219)
*n* = 100				
${\hat{\lambda }}_{GCV}$	SCAD	2.998(0.105)	1.500(0.106)	0.643(0.337)
	LASSO	2.907(0.121)	1.442(0.113)	0.256(0.211)
${\hat{\lambda }}_{BIC}$	SCAD	2.990(0.100)	1.499(0.097)	0.357(0.433)
	LASSO	2.894(0.126)	1.421(0.122)	0.216(0.184)

Overall, Tables 1 to 6 show that the nonzero estimates are unbiased regardless of the correlation structure. However, the unstructured correlation resulted in lower standard errors compared to the estimates based on an independent working correlation. The average number of zero coefficients is higher in the unstructured case. We notice a decrease in the mean ME when the sample size increases from 50 to 100 for both LASSO and SCAD. SCAD has a smaller mean ME than LASSO in all cases. We conclude that SCAD with BIC performs well.

### Case study

We now revisit the concrete slump test data set discussed in Section 1. From Fig 1, we see that slump (*Y*_1_) and flow (*Y*_2_) are highly correlated. We therefore used penalized GEE to perform the variable selection and parameter estimation. The resulting estimates are given in Tables 7 to 9. The second and third columns of the tables give the performance using penalized GEE with IWC for SCAD and LASSO. The fourth and fifth columns give the performance using penalized GEE with UWC. For the model selection procedures, both unweighted BIC and GCV were used to estimate the regression coefficients; their performance was similar. Therefore, we present only the results based on the unweighted BIC. Table 7 shows that SCAD with IWC selected 5 of the 7 covariates for slump (*Y*_1_), whereas LASSO with IWC selected 4 covariates. The difference is that LASSO omitted fine aggregate (*X*_7_). SCAD and LASSO with UWC obtained the same estimates for all the variables: they retained fine aggregate (*X*_7_) but forced fly ash (*X*_2_) and coarse aggregate (*X*_6_) to zero. Table 8 shows that both SCAD and LASSO with IWC selected fly ash (*X*_2_), water (*X*_4_), and coarse aggregate (*X*_6_) for flow (*Y*_2_), but SCAD and LASSO with UWC selected only fly ash (*X*_2_) and water (*X*_4_). The standard errors of the estimates is lower with UWC. Table 9 shows that LASSO with IWC selected all the covariates except coarse aggregate (*X*_6_) for CS (*Y*_3_), whereas the other methods dropped coarse aggregate (*X*_6_) and superplasticizer (*X*_5_).

**Table 7:** Estimates of regression coefficients for slump (*Y*_1_), with standard error in parentheses.

Variable	IWC		UWC	
	SCAD	LASSO	SCAD	LASSO
*X*_1_	–	–	–	–
	–	–	–	–
*X*_2_	-0.0297	-0.0375	–	–
	(0.0021)	(0.0013)	–	–
*X*_3_	-0.0061	-0.0098	-0.0023	-0.0023
	(0.0001)	(0.0010)	(0.0003)	(0.0003)
*X*_4_	0.0866	0.1222	0.0278	0.0278
	(0.0003)	(0.0025)	(0.0015)	(0.0015)
*X*_5_	–	–	–	–
	–	–	–	–
*X*_6_	-0.0011	-0.0017	–	–
	(0.0000)	(0.000)	–	–
*X*_7_	0.0070	–	0.0163	0.0163
	(0.0000)	–	(0.0000)	(0.0000)

**Table 8:** Estimates of regression coefficients for flow (*Y*_2_), with standard error in parentheses.

Variable	IWC		UWC	
	SCAD	LASSO	SCAD	LASSO
*X*_1_	–	–	–	–
	–	–	–	–
*X*_2_	-0.0529	-0.0715	-0.0169	-0.0169
	(0.0024)	(0.2544)	(0.0022)	(0.0022)
*X*_3_	–	–	–	–
	–	–	–	–
*X*_4_	0.2868	0.3341	0.2507	0.2507
	(0.0004)	(0.0077)	(0.0000)	(0.0000)
*X*_5_	–	–	–	–
	–	–	–	–
*X*_6_	-0.0033	-0.0121	–	–
	(0.0000)	(0.0031)	–	–
*X*_7_	–	–	–	–
	–	–	–	–

**Table 9:** Estimates of regression coefficients for compressive strength (*Y*_3_), with standard error in parentheses.

Variable	IWC		UWC	
	SCAD	LASSO	SCAD	LASSO
*X*_1_	0.1017	0.1032	0.0972	0.0972
	(0.0000)	(0.0000)	(0.0000)	(0.0000)
*X*_2_	0.0322	0.0337	0.0229	0.0299
	(0.0000)	(0.0000)	(0.0000)	(0.0000)
*X*_3_	0.0920	0.0931	0.0871	0.0871
	(0.0004)	(0.0003)	(0.0007)	(0.0007)
*X*_4_	-0.0866	-0.0802	-0.0494	-0.0494
	(0.0000)	(0.0000)	(0.0000)	(0.0000)
*X*_5_	–	0.0173	–	–
	–	(0.0000)	–	–
*X*_6_	–	–	–	–
	–	–	–	–
*X*_7_	0.0165	0.0174	0.0119	0.0119
	(0.0000)	(0.0000)	(0.0000)	(0.0000)

**Concrete slump test data with artificial binary response**. For illustration purposes, we create an artificial binary response variable to indicate whether or not a specimen can sustain a heavy load before distortion. For this analysis, we consider that concrete with a compressive strength below 35 is of poor quality. We therefore convert this continuous response to a binary based on the quality. Let *Y*_3_ = 1 if the compressive strength is above 35, and *Y*_3_ = 0 otherwise. The goal is to apply variable selection to model the correlated continuous and binary outcomes. The resulting estimates are given in Tables 10 to 12 (the columns of these tables are the same as those for Tables 7 to 9).

**Table 10:** Estimates of regression coefficients for slump (*Y*_1_), with standard error in parentheses.

Variable	IWC		UWC	
	SCAD	LASSO	SCAD	LASSO
*X*_1_	–	–	–	–
	–	–	–	–
*X*_2_	-0.0298	-0.0375	–	-0.0173
	(0.0017)	(0.0017)	–	(0.0000)
*X*_3_	-0.0061	-0.0098	-0.0042	-0.0071
	(0.0001)	(0.0016)	(0.0002)	(0.0002)
*X*_4_	0.0869	0.1222	0.0494	0.0753
	(0.0003)	(0.0041)	(0.0014)	(0.0097)
*X*_5_	–	–	–	–
	–	–	–	–
*X*_6_	-0.0011	-0.0017	–	–
	(0.0000)	(0.000)	–	–
*X*_7_	0.0070	–	0.0113	0.0073
	(0.0000)	–	(0.0001)	(0.0006)

**Table 11:** Estimates of regression coefficients for flow (*Y*_2_), with standard error in parentheses.

Variable	IWC		UWC	
	SCAD	LASSO	SCAD	LASSO
*X*_1_	–	–	–	–
	–	–	–	–
*X*_2_	-0.0529	-0.0715	-0.0192	-0.0514
	(0.0032)	(0.0672)	(0.0030)	(0.0013)
*X*_3_	–	–	–	–
	–	–	–	–
*X*_4_	0.2868	0.3341	0.2725	0.3171
	(0.0005)	(0.0086)	(0.0005)	(0.0088)
*X*_5_	–	–	–	–
	–	–	–	–
*X*_6_	-0.0034	-0.0121	-0.0041	-0.0104
	(0.0000)	(0.0011)	(0.0000)	(0.0005)
*X*_7_	–	–	–	–
	–	–	–	–

**Table 12:** Estimates of regression coefficients for binary compressive strength (*Y*_3_), with standard error in parentheses.

Variable	IWC		UWC	
	SCAD	LASSO	SCAD	LASSO
*X*_1_	0.0378	0.0448	0.0336	0.0431
	(0.0108)	(0.0463)	(0.0004)	(0.0039)
*X*_2_	0.0055	0.0077	0.0018	0.0057
	(0.0045)	(0.0108)	(0.0000)	(0.0016)
*X*_3_	0.0403	0.0471	0.0356	0.0451
	(0.0097)	(0.0430)	(0.0003)	(0.0037)
*X*_4_	-0.0361	-0.0483	-0.0292	-0.0416
	(0.0277)	(0.0410)	(0.0007)	(0.0091)
*X*_5_	–	–	–	–
	–	–	–	–
*X*_6_	-0.0089	-0.0104	-0.0082	-0.0098
	(0.0002)	(0.0008)	(0.0000)	(0.0001)
*X*_7_	–	0.0012	–	–
	–	(0.0009)	–	–

Table 10 shows that SCAD with IWC selected 5 of the 7 covariates for slump (*Y*_1_), whereas LASSO with IWC selected 4 covariates. The difference is that LASSO omitted fine aggregate (*X*_7_). These results are similar to the independent results in Table 7, which confirms the use of IWC. SCAD with UWC forced fly ash (*X*_2_) to zero whereas SCAD with IWC did not. LASSO with UWC selected the same variables as LASSO with IWC. Table 11 shows that all the methods selected fly ash (*X*_2_), water (*X*_4_), and aggregate (*X*_6_) for flow (*Y*_2_). Table 12 shows that all the methods except LASSO with IWC selected 5 covariates for the binary CS (*Y*_3_). The estimates obtained with UWC have lower standard errors.

## Conclusion

We have considered the selection of significant variables in multivariate multiple-response regression problems. We developed an extended GEE approach to take into account the correlation among the response variables. Our approach automatically and simultaneously selects the significant variables in high-dimensional models. We also proposed an efficient algorithm to implement the method. We performed many Monte Carlo simulations to assess the performance of the method for different sample sizes. The results showed that the methodology works well, especially when the SCAD penalty function is used together with the BIC tuning criterion. The estimates of ***β*** are unbiased regardless of the choice of correlation structure. We demonstrated the approach in a case study.
